# Deciphering the Crowd: Modeling and Identification of Pedestrian Group Motion

**DOI:** 10.3390/s130100875

**Published:** 2013-01-14

**Authors:** Zeynep Yücel, Francesco Zanlungo, Tetsushi Ikeda, Takahiro Miyashita, Norihiro Hagita

**Affiliations:** Intelligent Robotics and Communication Laboratories, Advanced Telecommunications Research Institute International, Kyoto 619-0288, Japan; E-Mails: zanlungo@atr.jp (F.Z.); ikeda@atr.jp (T.I.); miyasita@atr.jp (T.M.); hagita@atr.jp (N.H.)

**Keywords:** motion model, tracking, recognition

## Abstract

Associating attributes to pedestrians in a crowd is relevant for various areas like surveillance, customer profiling and service providing. The attributes of interest greatly depend on the application domain and might involve such social relations as friends or family as well as the hierarchy of the group including the leader or subordinates. Nevertheless, the complex social setting inherently complicates this task. We attack this problem by exploiting the small group structures in the crowd. The relations among individuals and their peers within a social group are reliable indicators of social attributes. To that end, this paper identifies social groups based on explicit motion models integrated through a hypothesis testing scheme. We develop two models relating positional and directional relations. A pair of pedestrians is identified as belonging to the same group or not by utilizing the two models in parallel, which defines a compound hypothesis testing scheme. By testing the proposed approach on three datasets with different environmental properties and group characteristics, it is demonstrated that we achieve an identification accuracy of 87% to 99%. The contribution of this study lies in its definition of positional and directional relation models, its description of compound evaluations, and the resolution of ambiguities with our proposed uncertainty measure based on the local and global indicators of group relation.

## 1. Introduction and Motivation

The observation of human behavior in public environments such as shopping malls, sport venues or stations is a common application. To increase our understanding of these data and utilize them more efficiently, we must associate attributes to individual pedestrians. The attributes of interest depend considerably on applications. For instance, resolving the social relation between customers such as mother-son, friends or couple is relevant in customer profiling [1]. Similarly, in intelligent environments, service quality can be improved by providing different services to clients by inferring their relation to their partners. Besides, in public environments, such as prisons or stadiums, recognizing the leader or the subordinates of groups is helpful for investigating aggressive or criminal activities [2,3].

However, the association of such attributes is considerably difficult due to the inherent contextual asperities and complex social relations. We propose treating this problem primarily by decomposing the entire crowd into smaller structures. In other words, we propose handling the crowd as a combination of social groups and single individuals. Once we obtain such a categorization, assigning social attributes is easier. We base our definition of *social groups* on the work of McPhail and Wohlstein [4], who regard a group as people engaged in a social relation to one or more pedestrians and move together toward a common goal.

**Figure 1.**
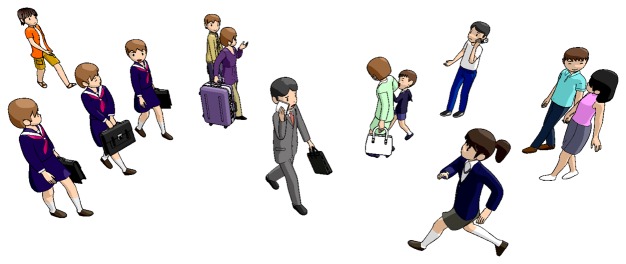
Which pedestrians are in a group? It is hard to tell from snapshots since traditional image based methods do not apply to surveillance footage. Trajectories are an important clue of group relation.

The detection of pedestrian groups is challenging from several perspectives. Figure 1 illustrates a scene, where the detection of group relations is not straightforward. This figure illustrates a scene from a public space, where friends and families are walking. Here, gender, clothing and age of the pedestrians are important cues indicating a social relation such as a couple or friends. Human cognition has evolved in such a way that these personal properties are identified easily in an unconscious manner. However, estimation of such cues from surveillance footage is not possible in most cases since traditional image based methods do not perform well for such recordings.

Therefore, we propose taking a closer look at the trajectories, namely the distribution of the displacements and scalar product of the velocity vectors. Based on these, we develop two explicit schemes for modeling the interaction among group members, in addition to two other schemes for modeling the interaction between groups and single pedestrians. The models are calibrated for different sorts of environments, group structures, and densities. With our proposed hypothesis testing scheme, we show that our method can resolve group relation to a considerable degree for various conditions.

The outline of the paper is as follows. Section 2 presents prominent works in this field, and Section 3 elaborates on the properties of the datasets employed in modeling and evaluation. Sections 4 and 5 discuss the motion models and the integration of individual indicators with the help of uncertainty measures. Finally, Section 6 presents our experimental results indicating stability, performance, sensitivity, and generalization issues in addition to a comparison with an earlier work in literature and an alternative decision scheme.

## 2. Background and Related Work

As smart environments spread, a vast amount of data is gathered, particularly from public spaces. The analysis of the crowd behavior in this sort of data is of great interest to numerous research fields such as crowd modeling and simulation, public space design, visual surveillance, and event interpretation [5]. In this section, we focus on previous works that interpret ambient information from a social relation perspective.

Human activity analysis bears numerous challenging traits [6]. For the solution of this problem, a social signaling standpoint is adopted by Cristani *et al.* [7], utilizing primarily the nonverbal cues of human behavior. Gatica-Perez gives a detailed overview of the nonverbal cues of small group relation, such as internal states, personality, and social relations [8]. Additionally, Costa demonstrates that group behavior presents distinctions in interpersonal distances depending on dominance, attraction, age similarity, and gender of the group members [9]. In the rest of this section, we refer to such complex features as the high-level cues of group relation. Such cues are specific to individuals. On the contrary, low-level cues involve features like spatial position, velocity or motion direction, which are not specific to individuals. We categorize low-level cues into two classes, linear and circular variables. Linear variables involve spatial position, trajectory shape, and the configuration of group members, while circular variables are composed of motion direction and the correlation of velocities.

Recently, the utilization of high-level cues has become a popular approach in the association of attributes to individuals, particularly in social network research. Several works address investigation of social relations based on such universally valid implicit cues as the age difference between parents and children or the opposite genders of heterosexual couples [10,11]. Some studies investigate kin relationships using photo albums that span a long time window of several years or even decades [12,13]. On the other hand, the proximity relation of faces on an image [14], clothing, or facial expressions [15] are used to estimate social relations.

For several contextual and practical reasons, these studies apply only to image domain and not to surveillance footage. First of all, in images from family albums or social network it is evident that the individuals appearing in the same image are related to each other. Then the question becomes resolving the type of relationship. However, the relation among pedestrians in a crowd is not obvious. Moreover, in video surveillance high-level cues are not available at all times.

To account for these challenging conditions, several studies propose integrating low-level and high-level cues. For instance, Ding *et al.* employ low-level cues in concept detection and define a Gaussian process based affinity learning for spotting social networks in theatrical movies and Youtube videos [16]. However, the appearance matrix relating the actors in a movie is derived from the script by searching for the names of the characters, which is not applicable in surveillance footage. By identifying the group structure, such behaviors as aggression or agitation are analyzed in [2]. Yu *et al.* assume that the 3D tracks of individuals and corresponding high-resolution face images are provided to investigate social groups and their organizations [3], which cannot be generalized to most other problems.

Compared with high-level cues, low-level ones are easier to derive. However, the analysis of group level activity based on low-level cues is profoundly integrated with stable multi-object tracking [1,17]. In other words, the occlusion arising from the group motion, which stands as a significant challenge at the first glance, can potentially be exploited for the enhancement of data association [18,19]. Namely, the search area is restricted based on the estimated future location of the objects from their past trajectories and motion models. Therefore, the dynamic models accounting for the collective locomotion behavior of pedestrians are proposed to improve tracking performance particularly against occlusions in [20–22].

By exploiting the low-level linear cues, several studies propose employing the contextual information provided by the configuration of groups to detect collective unusual behavior in public spaces. However, note that the problem of the resolution of group relations cannot be reduced to determining the similarity of trajectories [23]. The methods, which investigate similarity between individual trajectories, are mainly used in semantic scene modeling. They do not establish a relationship between simultaneously observed trajectories, which is the core of our problem [24,25]. Instead of finding the similarities between trajectories, Habe *et al.* propose finding interactions between trajectories to solve for mutual relationship between pedestrians. The influence that pedestrians exert on each other in the transition of motion states is investigated [26]. Floor control constitutes another commonly used low-level linear cue of collective human activities [27,28]. However, French *et al.* propose employing only the circular low-level cue of velocity correlation in a Bayesian framework and ignore the interpersonal distances [29]. In their framework, close proximity is not regarded as an indicator of group motion since it is claimed to be misleading in complex settings. Similarly, Calderara *et al.* omit the spatial relationships of trajectory points and focus on trajectory shapes [30]. Namely, they handle the problem from a circular statistics standpoint and cluster trajectories into similarity classes.

Yücel *et al.* suggest combining the linear and circular attributes [31–33]. In their framework, group relation is characterized by the distance between the moving parties and the alignment of their velocity vectors. Similarly, Ge *et al.* propose an algorithm to detect pedestrian groups through a bottom-up hierarchical clustering scheme based on locomotion similarities derived from an aggregated measure of velocity difference vectors and spatial proximity [34]. Similar to [34], Sandιkcι *et al.* propose to integrate the positional and directional cues in the resolution of group relations by defining similarity metrics for position, velocity, and direction, all of which in turn are expressed in a joint similarity matrix, followed by an agglomerative clustering approach [35]. Nonetheless, their motion models assume a very simple structure, which might not suffice to capture the distinctive attributes of group behavior. Bahlmann integrates linear and circular variables in a fairly different problem: online handwriting recognition [36]. Integration is achieved through an approximated wrapped Gaussian distribution, which only holds for data with low deviation, *i.e.*, *σ* < 1. Besides, this approach assumes that the probability density function of the linear variable is Gaussian. These two assumptions enable integration into multivariate semicircular wrapped distribution. However, neither holds for pedestrian trajectory data.

In addition to multi-object tracking and activity recognition, group models play an important role in such other fields as traffic analysis, evacuation dynamics, and the social sciences. Numerous works in pedestrians simulations are inspired by the social force model [37,38]. Lerner *et al.* describe a pedestrian simulation method, where a real world recording is employed to reflect behavioral complexity on individual level and group levels [39].

In light of these observations, we introduce a fundamental insight to collective pedestrian motion models by focusing on a short time interval and deriving low-level cues to infer the social relation. We aim to introduce a fundamental insight to collective pedestrian motion models. We relax the conditions defining group motion and provide a flexible means of identification for group relations. Since the final decision regarding group relations is based on the combination of positional and directional indicators, this problem is regarded as compound hypothesis testing. Various experiments prove that our proposed method effectively grasps the characterizing features of group relations and can recognize group activity with significantly high performance rates under varying environmental conditions and group configurations. Our paper makes the following contributions:

- Positional modeling accounting for dyadic as well as multi-partner groups;
- Directional modeling in both uniform and non-uniform environments;
- Integration of positional and directional indicators through compound hypothesis testing;
- Definition of local and global indicators and an uncertainty measure.

## 3. Datasets

Three publicly available datasets are employed in development and testing of the motion models, namely Caviar, BIWI Walking Pedestrians dataset, and APT Pedestrian Behavior Analysis dataset [20,40,41]. These are picked so as to effectively demonstrate the generalization capabilities of our proposed approach against varying environmental conditions and distinctions in group structure.

**Figure 2.**
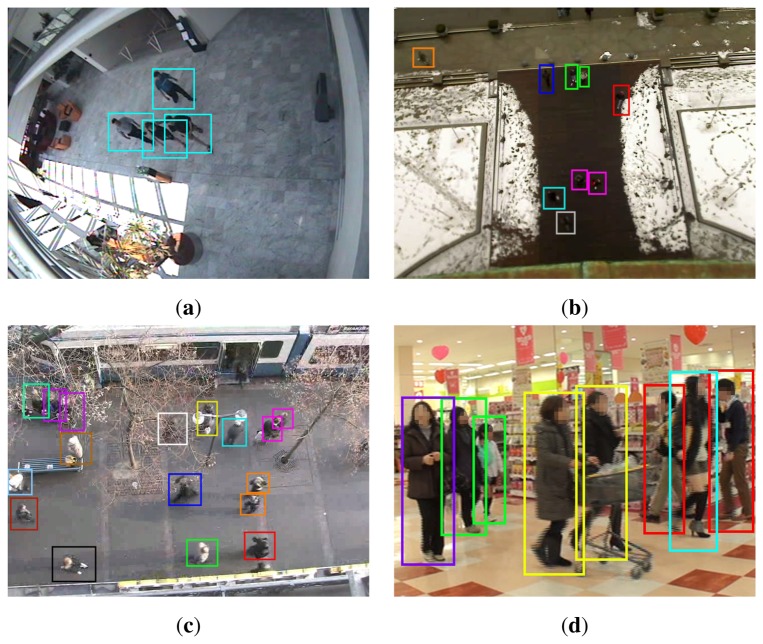
Experiment scenes from datasets (**a**) Caviar; (**b**) BIWI-ETH; (**c**) BIWI-Hotel and (**d**) APT. Pedestrians moving as a group are denoted with bounding boxes of the same color.

In Caviar dataset, five videos which are recorded from an oblique view over the entrance hall of a building involve group motion. The pedestrians present meeting and splitting behavior as well as uninterrupted group motion [40]. Although its size is quite moderate, Caviar dataset is considered in this study mainly due to the publicly available ground truth concerning groups, which provides a fair comparison with other methods. BrWI Walking Pedestrians dataset contains two sequences, BIWI-ETH and BIWI-Hotel, recorded from birds-eye view with a total of 650 tracks over 20 minutes [20]. The experiment scenes are the entrance of a building and a sidewalk. Due to the characteristics of these scenes, there is a dominant direction in the pedestrian flux (see Figure 2(b)). APT Pedestrian Behavior Analysis dataset is recorded in the entrance hall of a shopping center [41] (see Figure 2(c)). Unlike BIWI, such a prominent flow does not exist in any direction but a tendency to walk along a certain direction is noticed. Due to the homogeneous distribution of the flow, APT dataset is regarded as coming from a uniform environment.

Table 1 shows the total number of observed pedestrians and group sizes. The Caviar dataset involves a fairly small number of pedestrians. BIWI-ETH contains various multi-partner groups, whereas BIWI-Hotel and APT are composed of mainly dichotomous groups, who are often walking abreast. As the group size gets larger the possibility of abreast configuration decreases particularly in high pedestrian densities, *i.e.*, the groups may be bent forward or backward as well as arranged in a single file [42]. Among these sets, BIWI-ETH has the highest density followed by BIWI-Hotel, APT and Caviar, consecutively.

From Figure 2 and Table 1 the main differences between these sets are concluded to be the presence of preferred direction in BIWI-ETH and BIWI-Hotel against more homogeneous distribution in Caviar and APT and the frequent observation of multi-partner groups in BIWI-ETH against the dominance of dichotomous groups in BIWI-Hotel and APT. These variations are taken into consideration in the development of motion models.

Since this study proposes an identification method for groups of pedestrians rather than a tracking algorithm, we consider well-tracked trajectories and carry out our analysis to identify the pedestrian groups from these trajectories. For BIWI-ETH, BIWI-Hotel and ATR datasets, the trajectories which are obtained by state-of-the-art tracking algorithms, are publicly available [20,41,43,44]. For Caviar dataset, we performed manual annotation and estimated the homography matrix to map the annotated pixel coordinates to ground plane. The sampling period of trajectory points is 160 ms concerning BIWI-ETH and BIWI-Hotel sets and 100 ms concerning APT set. For Caviar dataset, the sampling rate is 200 ms. The group relations for all datasets are provided as ground truth [41,44,45]. Using these trajectories and ground truth values, a convenient formulation is offered in accordance with the characteristics of the environment and the group structure.

## 4. Modeling Indicators of Group Motion

The question addressed in this study is which parameters characterize group motion, how we can model them and determine whether two pedestrians belong to the same group or not. In what follows, we introduce the terminology used in the rest of this study and then describe our proposed models of the indicators of group motion.

**Figure 3.**
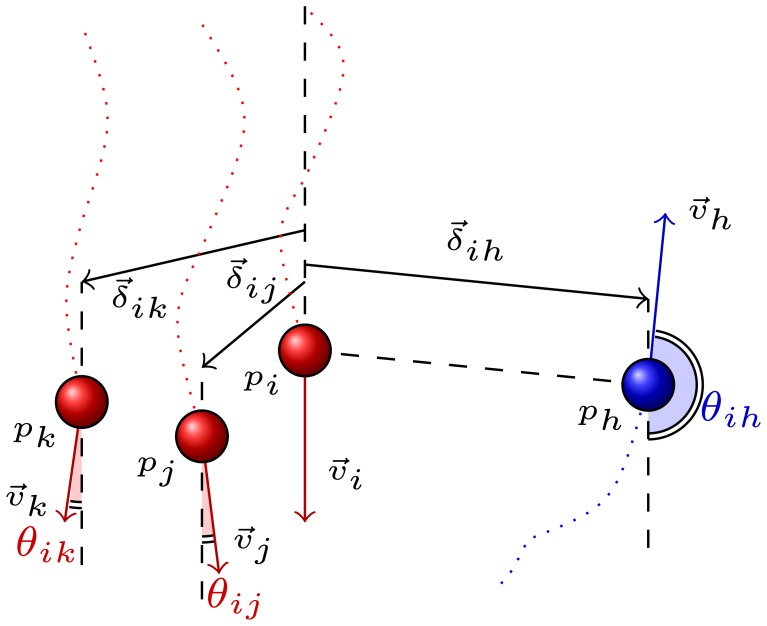
Pedestrians of same group are denoted with same color. Some positional and directional measures employed in identification of groups are illustrated in reference to *p_i_*.

We term any two pedestrians who are observed simultaneously as *a pair*. Suppose that the pairs who are engaged in a group relation such as {*p_i_*,*p_j_*} of Figure 3 constitute the set G, whereas the pairs who are not engaged in a group relation such as {*p_i_*,*P_h_*} comprise the complementary set Ḡ [4].

Based on the findings of [46], group motion is mainly characterized by positional indicators and directional indicators. We quantify positional indicators in terms of interpersonal distance, whereas directional indicators are defined based on motion directions. In explicit terms, the positional indicator of group motion is represented by Δ and is composed of a set of linear variables {*δ*}, where *δ* stands for the instantaneous distance between pedestrians (see Figure 3). On the other hand, the directional indicator, which is represented by Θ, is a set of circular variables, *i.e.*, angles between simultaneously observed velocity vectors {*θ*} (see Figure 3).

**Figure 4.**
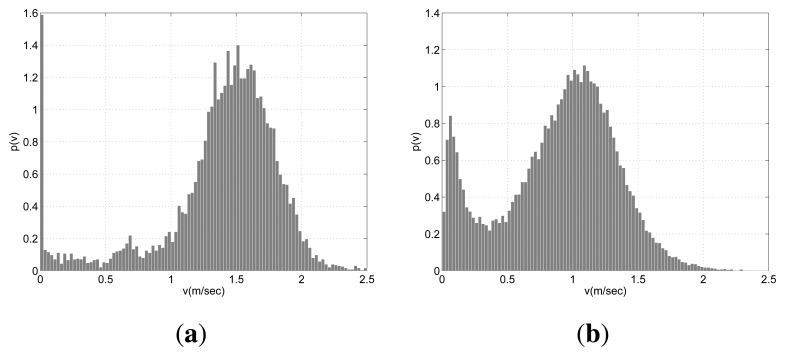
Velocity distribution concerning all pedestrians in (**a**) BIWI-ETH and (**b**) APT datasets.

Obviously, in order to define a meaningful value for *θ*, the pedestrians should be moving with a velocity larger than a reasonable threshold. We picked this value examining the distribution of velocity for all people in the environment (see Figure 4). In BrWI-ETH dataset the people who wait at the tram station have low velocities distributed more or less uniformly over 0 to 0.5 m/s. On the other hand, there are basically two peaks in velocity distribution for APT dataset. The first peak is entered around 0.1 m/s and it relates the people who are watching the shelves, whereas the second peak is centered around 1.2 m/s and it relates the people who walk steadily. Nevertheless, the number of these people is quite low compared with the steadily walking pedestrians. Thus, we picked 0.375 m/s as velocity threshold.

Since the velocity threshold is picked around the local minima of the velocity distribution separating the moving and stationary pedestrians, shifting the velocity threshold slightly would not affect a large number of pedestrians and thus not change the performance of the proposed method drastically. Moreover, the local minima observed in BIWI-ETH and APT datasets do not arise due the specific characteristics of these environments. According to Helbing *et al.*, at normal density the velocity of pedestrians is given by a normal distribution with an average of 1.34 m/s and a standard deviation of 0.26 m/s [47]. These values may change slightly according to the environment but putting the velocity threshold around 0.3 ∼ 0.5 m/s we will be sure to locate it at least 2*σ* from the peak [48].

Based on these definitions, each pair of pedestrians is represented by a set, which is composed of these two indicators {Δ,Θ}. Moreover, each of G and Ḡ is described by two models characterizing the positional and directional relations, *i.e.*, Δ_G_ and Θ_G_ or Δ_Ḡ_ and Θ_Ḡ_. The identification problem is deliberated with two different applications of the same approach in parallel, *i.e.*, investigating whether Δ ∼ Δ_G_ or Δ ∼ Δ_Ḡ_ and Θ ∼ Θ_G_ or Θ ∼ Θ_Ḡ_. The final decision is rendered based on the outcomes of these two, where the outcome implicating a lower uncertainty is preferred in case of ambiguities.

In our previous study we followed a similar strategy and proposed a simplistic method to identify group motion [31]. Ideally, the pedestrians involved in group motion are proposed to be in close proximity and have perfectly aligned velocity vectors. Since these ideal conditions are met seldom, certain thresholds are applied to account for the non-ideal nature of the behavior. In this manner, satisfactory performance rates are achieved. Nevertheless, explicit models are necessary to improve the performance and to make the method flexible in order to effectively adapt to different settings. To that end, the proximity and motion direction of pedestrians involved in a group relationship are investigated closely and a mathematical model is proposed for each of the relating probability density functions (pdf) in what follows.

### 4.1. Modeling Positional Indicators

The positional indicators are modeled based on the following assumptions. First an arbitrary reference frame is assigned to the observation environment. In addition, the probability of visiting each point in the environment is assumed to be equal,

(1) $$P({\text{p}}_{m})=P({\text{p}}_{n}),\forall {\text{p}}_{n},{\text{p}}_{n}\in A$$

where *P*(**p** *_m_*) denotes the probability of visiting point **p** *_m_* and *A* stands for the observation environment.

#### 4.1.1. Modeling Positional Indicators Regarding G

**Figure 5.**
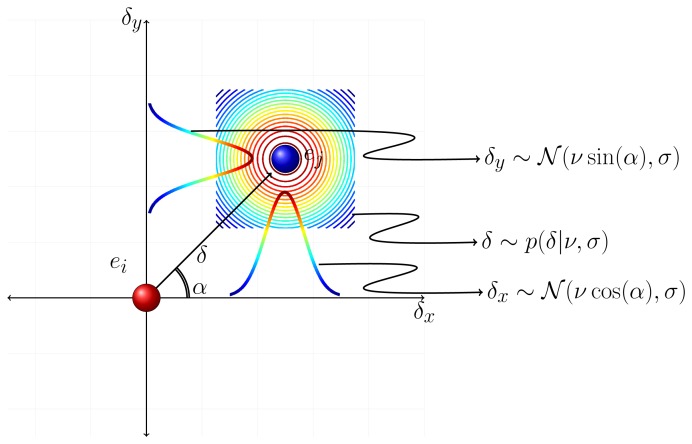
Distribution of *δ_x_*, *δ_y_* and *δ* regarding G.

Any displacement vector *δ⃗* can be decomposed into two components, *δ_x_* and *δ_y_*, where $\delta =\sqrt{\delta_{x}^{2}+\delta_{y}^{2}}$. Namely, *δ_x_* = *δ* cos(*α*) and *δ_y_* = *δ* sin(*α*), where *α* stands for the argument of *δ⃗* based on the chosen reference frame (see Figure 5). Since group members prefer to keep a comfortable distance of *ν* between each other, *δ_x_* and *δ_y_* are statistically independent normally distributed random variables,

(2) $$\begin{array}{c} \delta_{x}\sim N(\nu \cos(\alpha ),\sigma^{2})\\ \delta_{y}\sim N(\nu \sin(\alpha ),\sigma^{2}) \end{array}$$

Equation (2) implies that *δ* is distributed as a Rice distribution,

(3) $$p(\delta |\nu ,\sigma )=\frac{\delta }{\sigma^{2}}\exp\;\left(\frac{-\delta^{2}-\nu^{2}}{2\sigma^{2}}\right)\;I_{0}\;\left(\frac{\delta \nu }{\sigma^{2}}\right)$$

where *I*_0_ stands for the modified Bessel function of the first kind with order 0 [49].

This distribution is independent of the choice of reference frame. Of course, in the presence of a strong pedestrian flow along a certain direction, the distributions of *δ_x_* and *δ_y_* have different representations according to different choices of reference frame. This is due to the fact that *α* is determined by the major flow direction in such environments. In the presence of a major flow direction *α* is distributed in a non-uniform manner, which affects *δ_x_* and *δ_y_*. However, the distribution of *δ* given by Equation (3) is invariant to the orientation *α*. Thus, the distribution of *δ* is still given by Equation (3). This result obviously holds in the absence of any prominent direction such that *α* is a uniformly distributed circular random variable.

The unimodal formulation defined by Equation (3) provides a reasonable interpretation for the distance among members of a dichotomous group. However, multi-partner groups which are composed of three or more pedestrians, present more complex proxemics bearing a multimodal approach.

In order to have a better insight into the structure of multi-partner groups, we define *the degree of neighborhood* based on the configuration of the group members. Namely, the group structure is expressed in terms of a minimum spanning tree (MST). The degree of neighborhood concerning any two pedestrians is defined by the number of edges along the shortest path of the MST connecting them. According to this definition, {*p_i_*,*p_j_*} of Figure 3 has a degree of neighborhood that equals 1. In other words, they are are *first neighbors*, whereas {*p_i_*,*p_k_*} of Figure 3 are *second neighbors*.

In this framework, within multi-partner groups, the distance between first neighbors is modeled using the unimodal formulation of Equation (3). Assuming that the relative position of all first neighbors is given by the same function, *i.e.*, the distribution function for the position of first neighbors is the same within the group, the distance between *n^th^* order neighbors, *n* > 1, is modeled by the convolution of the unimodal model to the *n^th^* power. A multimodal framework, which is the linear combination of these *N* models is suggested to embrace the relation among members of a multi-partner group composed of *N* + 1 people. Namely,

(4) $$\Delta_{G}(\delta |\nu ,\sigma )=\sum_{n=1}^{N}K_{n}\Delta_{Gn}(\delta |\nu ,\sigma )$$

where *K_n_* is the observation frequency of *n^th^* neighborhood. The function Δ_G_*_n_* denotes the distribution between the *n^th^* neighbors and is equivalent to the convolution of Equation (3) to the *n^th^* power. It is suggested to restrict *N* ∈ {1, 2, 3}. Because large groups (of 5 or more people; tend to be arranged in complex configurations instead of abreast formation [42]. This limits the degree of neighborhood and eliminates the need to extend *N* over 3.

#### 4.1.2. Modeling Positional Indicators Regarding Ḡ

If any two simultaneously observed pedestrians are not engaged in a group relation, their relative locations at a particular instant are independent. This assumption, together with Equation (1), makes the problem equivalent to randomly selecting two points from a uniform distribution in the observation environment and measuring the distance between them. Suppose that the dimensions of the observation environment along the *x*− and *y*−axes are *D*. Then,

(5) $$p(\delta_{x})=\frac{2}{D}\left(1-\frac{\delta_{x}}{D}\right)$$

while the pdf concerning *δ_y_* is computed in the same manner. Assuming that *δ_x_* and *δ_y_* are independent, the relating joint pdf is resolved [50] as,

(6) $$p(\delta )=\{\begin{array}{ll} \frac{1}{D}2\delta (2\delta^{2}-4\delta +\pi ), & \text{if}\;0\leq \delta \leq D\\ \frac{1}{D}2\delta [4\sqrt{\delta^{2}-1}-(\delta^{2}+2-\pi )-4\tan^{-1}(\sqrt{\delta^{2}-1})] & \text{if}\;D<\delta \leq D\sqrt{2} \end{array}$$

This distribution describes *δ* regarding Ḡ in a large environment, *D* ≫ *c*, where *c* ≈ 400 mm stands for the width of the human body. However, it does not account for the constraint imposed by the physical dimensions of the pedestrians, that represent a minimum distance (cutoff) below which *5* cannot assume values. To account for this cutoff, *δ* is substituted with *δ*′ = *δ* − *c* and *p*(*δ*) is renormalized by replacing *D* with $D^{\prime }=D-c/\sqrt{2}$. Note that this distribution does not need to be calibrated since it only depends on the geometry of the observation area.

### 4.2. Modeling Directional Indicators

The directional indicator of group motion regarding any two pedestrians *p_i_* and *p_j_* is derived from their velocities. The scalar product of velocity vectors *υ⃗_i_* and *υ⃗_j_* is defined as,

(7) $$\frac{{\vec{\upsilon }}_{i}\cdot {\vec{\upsilon }}_{j}}{\left|{\vec{\upsilon }}_{i}\right|\left|{\vec{\upsilon }}_{j}\right|}=\cos(\theta_{ij})$$

where *θ* denotes the angle between these vectors (see Figure 3). The directional indicators of group motion are represented in terms of this angle *θ*.

The pairs in G, excluding those exhibiting behaviors like meeting, splitting or standing, are expected to have the direction of the velocity vectors aligned to a considerable degree, whereas the pairs in Ḡ do not present any correlation of direction. This suggests that the expected value of *θ* is 0 for both G and Ḡ. If *θ* were a linear random variable over (−∞, ∞), such a behavior could be approximated with a normal distribution of mean 0 and standard deviation *σ_θ_*. However, *θ* is a circular random variable defined over [−*π*, *π*] and, thus, it cannot be modeled in terms of a standard normal distribution.

Hence, the principles of directional statistics are invoked and the behavior of *θ* is modeled as a von Mises distribution [51], which is the circular analogue of the Gaussian distribution. The following is the explicit form of the von Mises distribution,

(8) $$p(\theta |\mu ,\kappa )=\frac{\exp(\kappa \cos(\theta -\mu ))}{2\pi I_{0}(\kappa )}$$

where *μ* denotes the mean value and *κ* is analogous of 1/*σ*^2^ of the normal distribution.

Note that the *θ* distribution relating G and Ḡ is described using the same function given by Equation (8), where the parameter *κ* enables modeling of different behaviors. In other words, for the pedestrian pairs in G, the distribution of *θ* is very localized around *μ* = 0 and *κ* ≫ 1. On the other hand, for the pedestrian pairs in Ḡ, the distribution is uniform if there is no prominent flow and *κ* → 0. Furthermore, in the presence of major flow *θ* has two peaks for each major flow, *i.e.*, one for pedestrians moving in the same direction and another for pedestrians moving in opposite directions. In that case, the distribution of *θ* regarding Ḡ is modeled as a linear combination of two von Mises distributions, one with *μ* = 0 and the other with *μ* = *π*. Even in this case, the distribution around a particular peak is expected to be larger than that of pairs in G.

## 5. Hypothesis Testing

The decision whether a pair belongs to G or Ḡ is carried out using a compound hypothesis testing scheme, as shown in Algorithm 1. Since G or Ḡ are mutually exclusive and complementary events, a decision can confidently be made as long as the individual indicators point to the same sort of group relation. In case of conflicts, a measure of uncertainty needs to be defined to resolve the final decision. In what follows we describe how the individual decisions are carried out and we define the uncertainty measures for resolving the final decision in case of contradictions.



**Algorithm 1:** Compound hypothesis testing.
 **Input:** Trajectories of pedestrian *p_i_* and simultaneously observed pedestrians {*p_j_*}, 1 ≤ *j* ≤ *J*. **Output:** The nature of group relation of *p_i_* with {*p_j_*} **for**
*j* ← 1 **to**
*J*
**do**  - Δ={|*δ⃗_ij_*|};  - Θ = {/(*υ^i^*,*υ^j^*)};  *- L^δ^*, *L^θ^*;/* Equation 9 */  **if** (*L^δ^* > 0) ∧ (*L^θ^* > 0);/* Equation 10 */  **then** {*p_i_,p_j_*} ∈ G;  **else if** (*L^δ^* < 0) ∧ (*L^θ^* < 0);/* Equation 10 */  **then** {*p_i_*,*p_j_*} ∈ Ḡ;  **else**   - Compute *ρ^δ^* and *ρ^θ^*;/* Equation 13 */   **if** [(Δ ∼ Δ_G_) ∧ (Θ ∼ Θ_Ḡ_) ∧ (*ρ^δ^* < 1/*ρ^θ^*)] ∨ [(Δ ∼ Δ_Ḡ_) ∧ (Θ ∼ Θ_G_) ∧ (*ρ^θ^* < 1/*ρ^δ^*)]   **then** {*p_i_*,*p_j_*} ∈ G;   **else** {*p_i_*,*p_j_*} ∈ Ḡ


In binary decisions, a likelihood ratio test is one way of determining the underlying model. Concerning Δ, the log-likelihood ratio of being in a group relation over not being in a group relation, *L^δ^*, is defined as,

(9) $$L^{\delta }=\log\left(\frac{\prod_{\forall \delta \in \Delta }\Delta_{G}(\delta |\nu ,\sigma )}{\prod_{\forall \delta \in \Delta }\Delta_{\bar{G}}(\delta )}\right)$$

The following is the decision based on *δ*,

(10) $$=\{\begin{array}{cc} \Delta \sim \Delta_{G} & L^{\delta }>0\\ \Delta \sim \Delta_{\bar{G}} & L^{\delta }<0 \end{array}$$

The decision based on *θ* is carried out in a similar manner through the log-likelihood ratio concerning Θ, *L^θ^*, computed in an analogous way to Equation (9).

As long as *L^δ^* and *L^θ^* have the same sign, a confident decision is made regarding the group relation (Algorithm 1 Lines 1 and 1). However, contradictions might arise. For example, when pedestrians cross next to each other, move along a flow, or go through passages, their relative position might become close or their velocity vectors might be aligned, independent of their social relation. One may argue that an intuitive way of resolving such cases is to pick the decision that implies a larger absolute value. However, we demonstrate in Section 6 that this straightforward approach is not capable of compensating for the effect of these misleading cues. Therefore, we devise an uncertainty measure.

Inspired by the Kullback-Leibler divergence, a reliability estimate is employed to quantify the uncertainty of individual decisions rendered through Equation (10) [52]. The Kullback-Leibler divergence of two distributions such as *P* and *Q* is defined as,

(11) $$D_{KL}(Q\|P)=\sum_{i}p(i)\log\left(\frac{\text{p}(i)}{q(i)}\right)$$

Note that this measure is not symmetric, *i.e.*, *D_KL_*(*Q*‖*P*) ≠ *D_KL_*(*P*‖*Q*). Thereby, mathematically speaking, it is not a distance measure but it quantifies the difference between two probability distributions. To have a common reference point, the divergence terms are computed with respect to the observed distributions. Hence, the divergences relating *8* with respect to G and Ḡ are defined as $D_{G}^{\delta }=D_{KL}(\Delta \|\Delta_{G})$ and $D_{\bar{G}}^{\delta }=D_{KL}(\Delta \|\Delta_{\bar{G}})$. Since these terms embrace all {*δ*} through the summation term in Equation (11), we call them *global indicators* of group motion.

However, *θ* relating G does not present a behavior as regular as *δ* of G. Thus, it is proposed to focus on its local characteristics so as to avoid the misleading temporal imperfections that might lead to a false similarity to Ḡ. Namely, the divergence term relating *θ* with respect to G is defined as,

(12) $$D_{G}^{\theta }(\Theta \|\Theta_{G})=\underset{\theta }{\max}\left\{\Theta (\theta )\log\left(\frac{\Theta (\theta )}{\Theta_{G}(\theta |\kappa )}\right)\right\}$$

where the divergence of *θ* with respect to Ḡ is computed in a similar manner. This equation implies that only the divergence value that indicates the maximum dissimilarity is accounted for. Thereby, it defines a local indicator of group motion.

A direct comparison of the divergence terms defined above is not possible since they are not defined in terms of comparable measures. To enable a comparison, two uncertainty measures are defined regarding each individual decision as the ratio of the concerning divergence values,

(13) $$\begin{array}{c} \rho^{\delta }=D_{G}^{\delta }/D_{\bar{G}}^{\delta }\\ \rho^{\theta }=D_{G}^{\delta }/D_{\bar{G}}^{\delta } \end{array}$$

The final resolution is determined by picking the decision with lower uncertainty (Algorithm 1 Line 1).

## 6. Experimental Results

This section discusses the performance of the estimated distributions in terms of a qualitative comparison, the stability of the model parameters with respect to varying training sets, the identification performance of groups, sensitivity, generalization, and improvement introduced by compound hypothesis testing over individual models and the method of [31] and maximum absolute log-likelihood ratio method.

### 6.1. Model Calibration

The models defined in Section 4 bear a number of parameters, which need to be tuned for different environments and group behaviors. For instance, the positional relation model regarding G, Δ_G_, given in Equation (4) requires the determination of *ν* and *σ*. Similarly, the directional relation models, Θ_G_ and Θ_Ḡ_, given in Equation (8) require calibration of *κ*.

For solving these model parameters, we propose shuffling the dataset and randomly selecting 10% of the pairs in G and 10% of the pairs in Ḡ. The squared error between the distributions of the positional and directional indicators concerning these randomly selected sets and the proposed models is minimized using a golden section search. Subsequently, the remaining 90% of the data is employed to evaluate of the proposed models. Section 6.2 presents the performance of this estimation scheme.

In our investigation of the stability of the model parameters, and the sensitivity of the model against varying training sets, this procedure is repeated by shuffling the dataset 50 times. Sections 6.3 and 6.4 report the performance metrics following such a validation scheme.

### 6.2. Estimated Distributions

**Figure 6.**
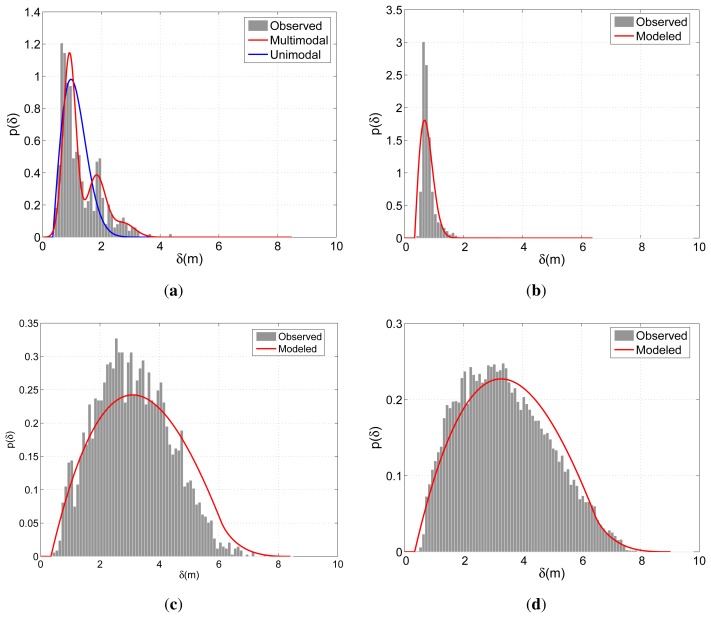
Observed and modeled distributions of *δ* regarding G for (**a**) BIWI-ETH and (**b**) APT. Figures (**c**) and (**d**) are organized similarly for Ḡ.

Figure 6 demonstrates the modeled and observed distributions of the positional indicators for a particular run of the calibration scheme described in Section 6.1. The observed distribution is expressed in terms of the histograms that relate the samples constituting the 90% of all observations. The model concerning Δ_G_ of BIWI-ETH is modeled with both unimodal and multimodal approaches. For this case, the multimodal approach in Equation (4) considers *N* to be 3. Since BIWI-ETH contains various multi-partner groups (see Table 1), the improvement of the multimodal approach over the unimodal approach can easily be observed in Figure 6(a). On the other hand, due to the dominance of the dichotomous groups in APT, the unimodal scheme provides satisfactory performance in modeling Δ_G_ concerning APT. For Δ_Ḡ_, fairly good results are obtained for both sets. The smoother shape of the observed distribution of APT is due to the larger number of observations compared with BIWI-ETH.

**Figure 7.**
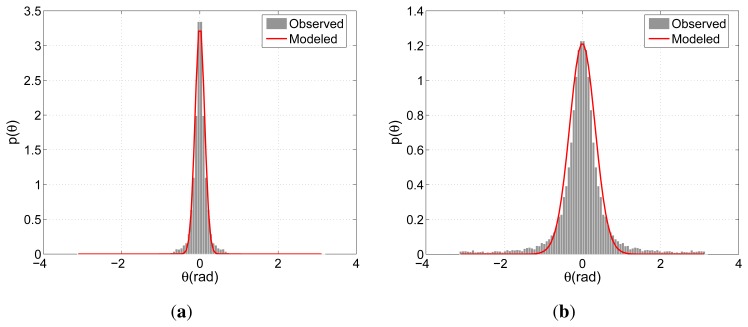

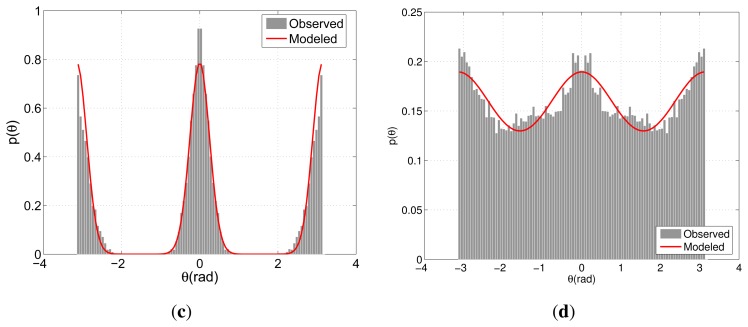
Observed and modeled distributions of *θ* regarding G for (**a**) BIWI-ETH and (**b**) APT Figures (**c**) and (**d**) are organized similarly for Ḡ.

Figures 7(a,b) illustrate the modeled and observed distributions of the directional indicators relating G. As expected, both models peak around 0, where the spread concerning APT is slightly larger than that of BIWI-ETH. This difference reflects the more regular motion pattern of the pedestrians due to fewer distractions in comparison with APT's shopping center environment. On the other hand, the models concerning Ḡ present a clear distinction arising from the different flow characteristics. Due to the lack of prominent flow direction, *θ* is distributed more evenly for APT and is concentrated around 0 and *π* for BIWI-ETH.

### 6.3. Stability of Parameters

Repeating the calibration method described in Section 6.1 50 times using a set of randomly selected samples that constitutes 10% of all the data, we obtain the statistics shown in Table 2.

The Δ_G_ models relating different datasets lead to similar values for *ν*, changing between 0.81 cm and 0.67 m with a fairly small variation within 0.06 m. Hall defines close phase personal distance to be between 46 cm and 75 cm and far phase personal distance to be between 76 cm and 120 cm [53]. Our findings are consistent with these values.

Regarding the *θ* models, the *κ* values relating G are always larger than those of Ḡ. As explained in Section 4.2, this indicates that the *θ* pattern concerning G is more structured than that of Ḡ. Nonetheless, the distinction becomes most clear in APT due to the lack of prominent flow direction. Moreover, the deviations of *κ* have quite insignificant values, provided that the sample set is large, as in BIWI-ETH and APT, whereas in BIWI-Hotel the deviation of *κ* regarding both G and Ḡ is higher relative to *κ* due to the reduced number of samples.

### 6.4. Performance and Sensitivity

Table 3 illustrates the performance of detecting the individual group relations of 50 runs of the proposed method together with the sensitivity of the identification rates. The overall success rates are all above roughly 85%, where the rates of G and Ḡ do not present any significant distinction between the different runs of the proposed method with respect to different datasets.

Since the group structure of multi-partner groups gets more complex, particularly in high pedestrian densities, it is not possible to provide stable statistics for the performance rates with respect to the degree of neighborhood [42]. This fact supports our unifying approach in modeling *δ* of G, where the different degrees of neighborhood are blended in Equation (4).

**Figure 8.**
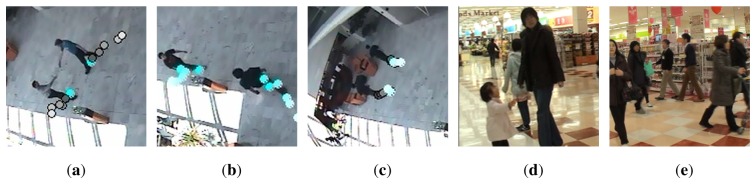

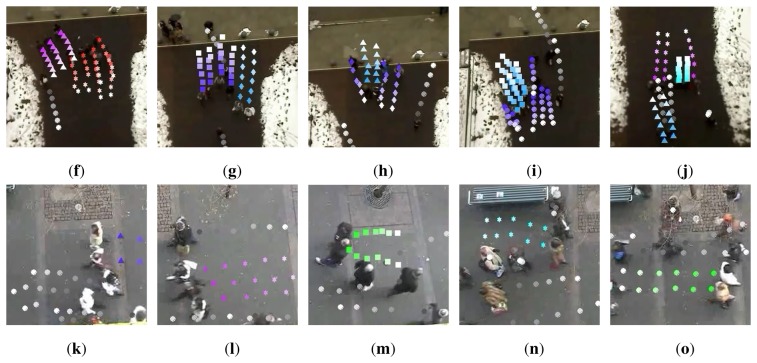
Pedestrians of same group are denoted with same marker and color, whereas pedestrians who do not belong to a group are denoted with gray circles. (**a,b,c**) Two pedestrians present meeting and splitting behavior; (**d**) Groups behave in a non-coherent manner; (**e**) Considerable occlusion; (**f,g**) Two groups move along same flow. Groups pass through each other moving (**h,i**) in opposite directions and (**j**) in same direction; (**k**) Unrelated pedestrians present group-like behavior; (**l,m**) Unrelated pedestrians follow similar trajectories with similar velocities to groups; (**n,o**) Waiting people introduce uncertainty.

Moreover, in multi-partner groups by applying a cross-check, the pedestrians, who are found to be in group relations to the same pedestrians independent of their degree of neighborhood, can be linked to each other. The detection rates regarding G increase to 100% by applying this cross-check. Figure 8 illustrates several examples of challenging cases from BIWI-ETH and BIWI-Hotel sets.

### 6.5. Comparison and Generalization

This section presents the performance rates based on the decisions of each individual indicator and ascertains that compound hypothesis testing improves the identification of group relations. Moreover, the alternative of hypothesis testing described in Section 5, where a decision is made in favor of the maximum absolute log-likelihood ratio, is applied and the superiority of our proposed method is verified. In addition, the detection performance of method of [31] is reported and it is ascertained that our proposed method outperforms it.

The improvement introduced by the integration of two observations through compound hypothesis testing as described in Section 5 is presented in Table 4. The improvement achieved by using both indicators (Δ + Θ), in comparison with using a single indicator (Δ or Θ), is presented in terms of the difference in performance rates of the individual decisions and performance rates after integration. It is observed that the numbers are often positive, which indicates that compound hypothesis testing provides an improvement over the individual models in almost every case.

The detection of G in Caviar is the only exception. Using the positional indicator Δ, a detection rate of 93.18% is achieved. Integrating positional and directional indicators, the detection rate decreases to 86.68%. This is due to the fact that the pedestrians in Caviar follow scenarios such as meeting and splitting, which cannot be determined using the directional indicators as explained in Section 4.2. The ground truth is given based on the video sequence, where visual cues are available. However, group relation is resolved using the indicators derived only from trajectory data. This implies that certain cues are not reflected such as gaze direction or body posture, whereas cues like position are still present. Therefore, it is not surprising that for describing behaviors like meeting and splitting, using only the positional indicator Δ results in a better performance than Δ + Θ.

Table 5 illustrates the performance rates of the model of [31] for pairs in G, pairs in Ḡ and all pairs. In BIWI-ETH, which involves a non-uniform environment with high pedestrian density, Reference [31] has a positive bias for Ḡ, which misleadingly increases the overall detection rate to 95.05%. However, G, which is observed less often than Ḡ is only detected by a 65.52% success rate. In BIWI-Hotel, which involves a dominant flow direction environment with low pedestrian density, the identification rates of [31] and the proposed method are comparable. In APT, Reference [31] detects both G and Ḡ with roughly 9% lower rates than our method.

In Section 5, it is mentioned that a straightforward way of dealing with conflicting decisions is to pick the decision that implies a larger absolute value. The identification rates achieved by selecting the decision with a higher absolute log-likelihood ratio instead of applying compound hypothesis testing is presented in Table 5. Although the overall performance rates seem close to the proposed method, the detection rates of G are considerably lower than those of Ḡ. In other words, this approach has a positive bias for Ḡ. Therefore, our proposed method proves to have no bias in favor of a particular class, which implies a fair distinction of group relation.

## 7. Conclusions

Positional and directional models are proposed for identification of pedestrian groups in crowded environments together with a compound evaluation scheme. Different environmental characteristics are accounted for in addition to varying group structures. Our results indicate that our proposed models grasp the characterizing features of different environmental settings and varying patterns of group relations. Moreover, the model parameters are shown to be stably derived from a small set of data. In addition, the group relations are illustrated to be identified with satisfactorily high rates. The efficacy of compound evaluations is verified by a comparison with individual decisions as well as with another method in the literature. Finally, our contributions are listed as improvements in positional and directional models to adjust to different environments and group structures, the description of compound evaluations and the comparison of the models, and the resolution of ambiguities with our proposed uncertainty measure based on the local and global indicators of group relations.

## Figures and Tables

**Table 1.** Specifications of datasets.

	**Duration**	**Group size**					**Total # of pedestrians**

		**2**	**3**	**4**	**5**	**6**	
Caviar	1′11″	5	-	1	-	-	17
BIWI-ETH	8′38″	38	10	6	1	4	360
BIWI-Hotel	12′54″	38	3	-	-	-	223
APT	30′00″	128	8	-	-	-	531

**Table 2.** The mean values and standard deviations of *ν*, *σ* and *κ* over the 50 runs.

		**Caviar**	**BIWI-ETH**	**BIWI-Hotel**	**APT**
Δ_G_(*δ*|*ν,σ*)	*ν*	0.81 ±0.04	0.76 ±0.06	0.67 ±0.03	0.71 ±0.02
	*σ*	0.33 ±0.07	0.22 ±0.05	0.14 ±0.03	0.13 ±0.02

Θ_G_(*θ*|*κ*)	*κ*	6.36 ±1.15	69.53 ±9.18	164 ±40.25	9.59 ±2.11

Θ_Ḡ_(*θ*|*κ*)	*κ*	0.32 ±0.39	15.03 ±1.38	36.29 ±9.18	0.89 ±0.13

**Table 3.** Performance rates of the proposed method.

	**G**(%)	**Ḡ**(%)	Total(%)
Caviar	86.68 ± 0.33	94.36 ± 0.23	87.82 ± 0.32
BIWI-ETH	85.62 ± 0.00	91.15 ±0.00	90.51 ±0.00
BIWI-Hotel	95.89 ± 0.33	96.77 ±1.61	96.57 ±0.51
APT	94.77 ±0.15	99.84 ±0.10	99.10 ±2.76

**Table 4.** Improvement introduced by compound evaluation over individual decisions.

		**G**(%)	Ḡ(%)	Total(%)
Caviar	Δ → Δ + Θ	−6.5	22.4	−2.7
	Θ → Δ + Θ	4.96	3.89	4.77

BIWI-ETH	Δ → Δ + Θ	3.03	0.36	0.47
	Θ → Δ + Θ	13.62	2.65	3.18

BIWI-Hotel	Δ → Δ + Θ	0.06	0.04	0.04
	Θ → Δ + Θ	0.33	0.04	0.07

APT	Δ → Δ + Θ	5.74	0.56	3.14
	Θ → Δ + Θ	8.05	9.66	8.87

**Table 5.** Performance comparison of the proposed method to the method of [31] and method of maximum absolute log-likelihood ratio.

	**Proposed method** (%)			**Method of** [31] (%)			**Maximum absolute log-likelihood ratio** (%)		
		
	**G**	**Ḡ**	Total	**G**	**Ḡ**	Total	**G**	**Ḡ**	Total
Caviar	86.68	94.36	87.82	57.50	94.83	63.17	55.88	86.28	60.49
BIWI-ETH	85.62	91.15	90.51	65.52	97.23	95.05	58.81	96.16	88.79
BIWI-Hotel	95.89	96.77	96.57	97.87	96.21	96.25	88.75	98.18	96.03
APT	94.77	99.84	99.10	88.08	89.81	88.33	86.72	99.65	97.77
